# Supplementary material for: Purchase, consumption, and ownership of chickens and chicken products among households in Maputo, Mozambique: A cross-sectional study
Source: One Health. 2024 Nov 22;19:100943. doi: 10.1016/j.onehlt.2024.100943 (PMC11626821; doi:10.1016/j.onehlt.2024.100943)
Supplement: Supplementary file 1 — Supplementary Tables and Supplementary Methods [file mmc1.docx]

# SUPPLEMENTARY TABLES

### Supplementary Table 1. Multi-stage cluster sampling strategy in Maputo, Mozambique.

| **District** | **Population** | **Percentage of the total population of Maputo** | **Number of clusters of 20 households** | **Number of neighborhoods** | **Name of sampled neighborhoods** |
| --- | --- | --- | --- | --- | --- |
| KaMavota | 293,766 | 29% | 8 | 4 | 3 de Fevereiro  Costa do Sol  Mahotas  Hulene B |
| KaMaxakeni | 223,936 | 22% | 6 | 3 | Urbanização  Maxaquene A  Maxaquene C |
| KaMubukwana | 304,524 | 31% | 8 | 4 | Malhazine  Zimpeto  Bagamoyo  Inhagoia B |
| KaTembe | 20,629 | 2% | 1 | 1 | Chali |
| Nlhamankulu | 155,462 | 16% | 4 | 2 | Chamanculo D  Aeroporto A |
| **Total (City of Maputo)** | **998,317** | **100%** | **27** | **14** |  |

### Supplementary Table 2. Source of live chickens, chicken meat, eggs, and chicken litter compost among households that purchased these products in the past week of the survey date in Maputo, Mozambique in May-June 2021 (unweighted statistics).

| **Source** | **Broiler chicken meat** | **Live broiler chickens** | **Live indigenous chicken** | **Eggs** | **Chicken litter compost** |
| --- | --- | --- | --- | --- | --- |
|  | **(N=250)** | **(N=49)** | **(N= 6)** | **(N=263)** | **(N=58)** |
| Markets | 43 (17.2%) | 9 (18.4%) | 1 (16.7%) | 43(16.3%) | 1 (1.7%) |
| Supermarkets | 7 (2.8%) | 0 (0%) | 0 (0%) | 10 (3.8%) | 0 (%) |
| Corner stores | 141 (56.4%) | 0 (0%) | 2 (33.3%) | 162 (61.6%) | 0 (%) |
| Family/friends/neighbors | 4 (1.6%) | 5 (10.2%) | 3 (50%) | 13 (5.0%) | 13 (22.4%) |
| Directly from farmers | 45 (18.0%) | 32 (65.3%) | 0 (0%) | 22 (8.4%) | 23 (39.7%) |
| Directly from layers that I own | 1 (0.4%) | 1 (2.0%) | 0 (0%) | 6 (2.3%) | 2 (3.4%) |
| Directly from broiler chickens that I own | 8 (3.2%) | 2 (4.1%) | 0 (0%) | 1 (0.4%) | 14 (24.1%) |

### Supplementary Table 3. Source of live chickens, chicken meat, eggs, and chicken litter compost among households that purchased these products in the past week of the survey date in Maputo, Mozambique in May-June 2021 (weighted statistics^1^ with percentages for total households (N=175,143)).

###

| **Source** | **Broiler chicken meat** | **Live broiler chickens** | **Live indigenous chicken** | **Eggs** | **Chicken litter compost** |
| --- | --- | --- | --- | --- | --- |
|  | **(N=78,436)** | **(N=14,986)** | **(N=1,950)** | **(N=81,432)** | **(N=17,891)** |
| Markets | 13,820 (7.9%) | 2,903 (1.7%) | 316 (0.2%) | 13,054 (7.5%) | 327 (0.2%) |
| Supermarkets | 2,289 (1.3%) | 0 (0%) | 0 (0%) | 3,221 (1.8%) | 0 (0%) |
| Corner stores | 44,734 (25.5%) | 0 (0%) | 636 (0.4%) | 50,119 (28.6%) | 0 (0%) |
| Family/friends/neighbors | 1,301 (0.7%) | 1,613 (0.9%) | 998 (0.6%) | 4,010 (2.3%) | 4,223 (2.4%) |
| Directly from farmers | 13,267 (7.6%) | 9,506 (5.4%) | 0 (0%) | 6,869 (3.9%) | 6,903 (3.9%) |
| Directly from layers that I own | 316 (0.2%) | 316 (0.2%) | 0 (0%) | 1,941 (1.1%) | 640 (0.4%) |
| Directly from broiler chickens that I own | 2,619 (1.5%) | 648 (0.4%) | 0 (0%) | 316 (0.2%) | 4,232 (2.4%) |

^1^Survey weighting was conducted using district level population data. The design weight for each participating household was the inverse of their probability of selection in the corresponding district.

### Supplementary Table 4. Source of live chickens, chicken meat, eggs, and chicken litter compost among households that purchased these products in the past week of the survey date in Maputo, Mozambique in May-June 2021 (unweighted statistics with percentages for total households (N=570)).

| **Source** | **Broiler chicken meat** | **Live broiler chickens** | **Live indigenous chicken** | **Eggs** | **Chicken litter compost** |
| --- | --- | --- | --- | --- | --- |
|  | **(N=250)** | **(N=49)** | **(N= 6)** | **(N=263)** | **(N=58)** |
| Markets | 43 (7.5%) | 9 (1.6%) | 1 (0.2%) | 43 (7.5%) | 1 (0.2%) |
| Supermarkets | 7 (1.2%) | 0 (0%) | 0 (0%) | 10 (1.8%) | 0 (%) |
| Corner stores | 141 (24.7%) | 0 (0%) | 2 (0.4%) | 162 (28.4%) | 0 (%) |
| Family/friends/neighbors | 4 (0.7%) | 5 (0.9%) | 3 (0.5%) | 13 (2.3%) | 13 (2.3%) |
| Directly from farmers | 45 (7.9%) | 32 (5.6%) | 0 (0%) | 22 (3.9%) | 23 (4.0%) |
| Directly from layers that I own | 1 (0.2%) | 1 (0.2%) | 0 (0%) | 6 (1.1%) | 2 (0.4%) |
| Directly from broiler chickens that I own | 8 (1.4%) | 2 (0.4%) | 0 (0%) | 1 (0.2%) | 14 (2.5%) |

# SUPPLEMENTARY METHODS

## Modules and questions in the survey

We first gave a consent to at least one adult household member to participate in the survey. Next, we conducted the COVID-19 screening and postponed a household visit if a household failed to pass the screening questions.

There were seven modules in the survey, followed by an observational component. Information was collected for each type of poultry products (meat and egg) as well as the following type of chickens: broiler chickens (i.e., chickens specifically bred to provide meat), layer chickens, and indigenous chickens (i.e., local chickens, native to Mozambiquie). For households with children under five years of age, we specifically asked about the corresponding information for children.

In the first module (Module A), we asked about household demographics, such as the number of household members and the number of children under five.

The second module (Module B) was about the purchase and consumption of chicken meat and eggs. We asked whether households purchased chicken meat and eggs in the previous week of the survey date, and if yes, asked about the location/source where they purchased them. We asked whether households consumed chicken meat (broiler, indigenous) and/or eggs at any setting, including but not limited to the home and restaurants, and if yes, how many days in the past week they ate them. For households with children under five, we asked these questions for children as well. We also asked where households store uncooked chicken meat.

The third module (Module C) was about the history of raising chickens for sale. We asked whether households previously raised chickens for sale at their house or compound, and if yes, whether they had a chicken coop and how many flocks or groups of birds they produced last year. If household reported that they used chicken litter as bedding, we asked what they usually do with the used chicken litter (e.g., throw it away, reuse it for another flock, repackage it to sell, apply it to garden).

The fourth module (Module D) was about the current ownership of live chickens (layers, broilers, and indigenous chickens). We asked whether households currently raise live chickens for sale or consumption, and if yes, how many live chickens they currently own and where they most often keep them during the day and at night. We then asked whether households purchased live chickens for their own consumption or use in the previous week from the survey date, and if yes, we asked about the location/source of these live chickens. For households with children under five, we asked whether children help take care of live chickens. We also asked what measures, if any, households take to separate chickens from children (e.g., corralling chickens).

The fifth module (Module E) was about chicken litter compost. We asked if households had a garden, and if yes, whether they apply chicken litter compost to the garden. We also asked where they purchase or get chicken litter compost and if children help apply chicken litter compost to their garden.

The sixth module (Module F) was about water, sanitation and hygiene (WASH), and the last module (Module G) was about COVID-19. We asked whether households had any negative effect from the pandemic in their households, what kind of aid they had received if any, what kind of changes they experienced during the pandemic (e.g., frequency of market visits), hygiene practice and other personal prevention measures during the pandemic, and their perspectives on COVID-19.

In the end of the household visit, enumerators conducted an observation related to hygiene. They observed handwashing facilities, products for general cleaning, and where households keep chickens.
